# Bacterial Consumption of T4 Phages

**DOI:** 10.3390/microorganisms9091852

**Published:** 2021-08-31

**Authors:** Jean-Jacques Godon, Ariane Bize, Hoang Ngo, Laurent Cauquil, Mathieu Almeida, Marie-Agnès Petit, Olivier Zemb

**Affiliations:** 1 INRAE, Univ Montpellier, LBE, 11100 Narbonne, France; 2 PRocédés biOtechnologiques au Service de l’Environnement, INRAE, Université Paris-Saclay, 92761 Antony, France; ariane.bize@inrae.fr (A.B.); hoang.ngo@inrae.fr (H.N.); 3 GenPhySE, INRAE, Université de Toulouse, 31320 Castanet-Tolosan, France; laurent.cauquil@inrae.fr; 4 MGP, INRAE, Université Paris-Saclay, 78350 Jouy-en-Josas, France; Mathieu.Almeida@inrae.fr; 5 AgroParisTech, Micalis Institute, INRAE, Université Paris-Saclay, 78350 Jouy-en-Josas, France

**Keywords:** bacteriophage, Aeromonas, stable isotope probing

## Abstract

The bacterial consumption of viruses not been reported on as of yet even though bacteria feed on almost anything. Viruses are widely distributed but have no acknowledged active biocontrol. Viral biomass undoubtedly reintegrates trophic cycles; however, the mechanisms of this phase still remain unknown. ^13^C-labelled T4 phages monitor the increase of the density of the bacterial DNA concomitant with the decrease of plaque forming units. We used ^12^C T4 phages as a control. T4 phage disappearance in wastewater sludge was found to occur mainly through predation by *Aeromonadacea*. Phage consumption also favours significant in situ bacterial growth. Furthermore, an isolated strain of *Aeromonas* was observed to grow on T4 phages as sole the source of carbon, nitrogen, and phosphorus. Bacterial species are capable of consuming bacteriophages in situ, which is likely a widespread and underestimated type of biocontrol. This assay is anticipated as a starting point for harnessing the bacterial potential in limiting the diffusion of harmful viruses within environments such as in the gut or in water.

## 1. Introduction

For any type of bacteria, the presence of viruses may present a significant opportunity for feeding. Indeed, viruses represent 0.2 gigatons of carbon on Earth [1]. For example, the major capsid protein of the T4-like bacteriophage family is one of the most prevalent proteins in the biosphere [2]. Therefore, phages represent a major potential carbon source that bacteria may tap into. Furthermore, viruses are also a potential source of phosphorus [3].

No bacterium preying on viruses have been described even though bacterial extracellular proteases are able to degrade certain bacteriophages in anaerobic wastewater treatment plants, in pure cultures [4], and in soil [5]. In seawater, the only reported biotic pressure arises from marine ciliates that have been co-incubated with viruses and bacteria [6]. This observation is also supported by the recent discovery of viral DNA in free-living eukaryotic cells [7]. 

Here, we show that specific bacteria can indeed degrade T4 bacteriophages in situ, and we confirm this observation in pure culture.

## 2. Materials and Methods

### 2.1. Preparation of the ^13^C-Labeled T4 Bacteriophages

T4-phage particles labeled with ^13^C were produced on *Escherichia coli* B cells (DSM 613) grown in M9 minimal medium with ^13^C-glucose as the sole carbon source. The M9 medium was prepared using M9, Minimal Salts, 5X (Sigma-Aldrich, St. Louis, MI, USA), by adding MgSO_4_ (Sigma-Aldrich) and CaCl_2_ (Sigma-Aldrich) at final concentrations of 1 mM and D-Glucose at a final concentration of 10 g/L. Moreover, additional salts were added to favor phage adsorption (a solution of CaCl_2_ 0.5 M and MgCl_2_ 1M diluted 1000 times in the culture medium). More precisely, starting from an *E. coli* stock of cells frozen in LB and glycerol, two successive overnight pre-cultures were grown in LB medium (LB broth, Fisher). Subsequently, 5 × 20 mL of M9 minimal medium containing D-Glucose-^13^C6 as the sole carbon source (10 g/L) were each inoculated with 20 µL of the second *E. coli* pre-culture; approximately 1500 T4-phage particles (DSM 4505, in PFU) were added. Finally, 20 µL of a solution containing 0.5M CaCl_2_ and 1M MgCl_2_ was also added in each case to favor phage adsorption.

After 30 h of incubation at 37 °C under agitation, the T4 phage particles were collected: the cultures were centrifuged for 15 min at 5000× *g* and at a temperature of 10 °C. The supernatants were collected and filtered with 0.22 µm pore-sized PES filters (Millipore, Burlington, MA, USA). They were subsequently incubated overnight in 8% *w/v* PEG 6000 and 0.5 M NaCl solution at 4 °C to precipitate viral particles. The supernatants were centrifuged at 20,000× *g* for 30 min at 4 °C. The pellets were suspended in SM buffer (100 mM NaCl, 8 mM MgSO_4_, 50 mM Tris pH 7.5) and were centrifuged once more at 20,000× *g* for 4h at 4 °C. The viral particles were finally suspended in 1.4 mL of SM buffer and were stored at 4 °C before use (Appendix C Figure A3 for details). To obtain unlabeled T4 phage particles, the same procedure was used, except unlabeled glucose was employed in the M9 minimal medium.

### 2.2. Incubation of ^13^C-Labeled T4 Bacteriophages with Aerobic Sludge

A 100 mL sample was taken from an aerobic wastewater treatment plant and was stabilized without the addition of substrate for one week at room temperature.

The T4 phages were incubated at 20 °C with 200 µL of the initial sample from the wastewater treatment plant under 50 rpm agitation. The initial concentration in the ^13^C bottle was 2.24 × 10^10^ T4 phages in 5 mL, i.e., 4.48 × 10^9^ T4 phages/mL. The PFU titers of the obtained; ^13^C- and unlabeled T4 stock solutions were determined on a bacterial lawn of *E. coli* cells (DSM 613) using the soft-agar overlay technique. More precisely, 5 µL of T4 phage stock solutions and 10-fold serial dilutions of those solutions were plated on a soft layer containing 7.5 g/L of Agar and *E. coli* cells (DSM 613) that had been pre-cultured in LB medium and in LB-Agar plates (15 g/L of Agar, Sigma-Aldrich). After a short drying period, the Petri dishes were incubated at 37 °C over 24 h in static conditions. The PFU titers were determined by counting the visible plaques and by calculating the concentration in the original stock solutions.

### 2.3. DNA Extraction and 16S rDNA Analysis

The three samples (the initial sample from the wastewater treatment plant, the ^12^C bottle after 24 h of incubation, and the ^13^C bottle after 24h of incubation) were used for ribosomal 16S DNA sequencing and analysis. After the addition of 3 × 10^5^ copies of internal standard [8], bead beating lysed the microbial cells, and the DNA was purified using the ZR-96 Soil Microbe DNA kit according to the manufacturer’s description (Zymo Research, Irvine, CA, USA). The V4-V5 region was amplified from purified genomic DNA with the primers 515F (5′-CTTTCCCTACACGACGCTCTTCCGATCTGTGYCAGCMGCCGCGGTA) and 928R (5′-GGAGTTCAGACGTGTGCTCTTCCGATCTCCCCGYCAATTCMTTTRAGT) using 30 amplification cycles with an annealing temperature of 65 °C (an amplicon of 510 bp, although length varies depending on the organisms). Because the Illumina MiSeq technology enables paired 300-bp reads, the ends of each read overlap and can be stitched together to generate extremely high-quality, full-length reads of the entire V4-V5 region in a single run. Single multiplexing was performed using a homemade 6 bp index, which were added during a second PCR with 12 cycles using a forward primer (AATGATACGGCGACCACCGAGATCTACACTCTTTCCCTACACGAC) and a reverse primer (CAAGCAGAAGACGGCATACGAGAT-index-GTGACTGGAGTTCAGACGTGT). The resulting PCR products were purified and were loaded onto the Illumina MiSeq cartridge according to the manufacturer’s instructions. The quality of the run was checked internally using PhiX control as recommended by manufacturer, and then each pair-end sequence was assigned to its sample with the help of the previously integrated index. Each pair-end sequence was assembled using Flash software [9] using at least a 10bp-overlap between the forward and reverse sequences. The absence of contamination was checked with a negative control during the PCR (water as the template). The quality of the stitching procedure was controlled using four bacterial samples that are run routinely in the sequencing facility in parallel to the current samples.

The resulting sequences were analyzed using the DADA2 pipeline (maxN = 0, truncQ = 2, trimLeft = c(17,17), pool = “pseudo”) [10] with the Silva 138 database [11]. Chimeras were removed by means of the DADA2 using the consensus method. Normalisation was performed using the internal standard, and the total bacterial 16S rDNA was measured using qPCR (see below).

### 2.4. Detailed Calculations of the ^13^C Mass Balance

This paragraph explains the mass balance in detail. For a simpler explanation, here, we will focus on the calculations for the Amplicon Sequence Variant ASV1 (because it corresponds to the Aeromonas_isolate_007 that we used to confirm the consumption of T4 phages in pure culture).

STEP1: using qPCR, we determined the absolute abundance of total bacteria in the ^13^C bottle at the beginning of the experiment and at the end of the experiment. This could be conducted because we spiked 3 × 10^5^ copies of a synthetic DNA standard to 200 µL of the initial sample [8], which we extracted immediately and quantified using 16S the universal primers 515F-928R with the Illumina adapters. We also quantified the spiked synthetic DNA standard by qPCR [8]. The ratio between the internal standard and the total bacterial 16S rDNA indicates the number of 16S rDNA copies in the extraction tube from the ^13^C bottle independently from the DNA recovery yield, which was estimated to be 2.35 × 10^8^ copies of 16S rDNA. For an accurate mass balance, we also considered the fact that 240 µL were removed for the PFU measurements during the experiment, so we estimated that 2.47 × 10^8^ copies of 16S rDNA would have been present at the end of the experiment in the ^13^C bottle if no sampling had been performed.

STEP2: Using the absolute abundance of total 16S rDNA from STEP1 and 16S barcoding, we converted the proportion of each ASV into the absolute abundance of each ASV at the beginning and at the end of the experiment, thereby estimating the number of 16S rDNA copies produced during the experiment for each ASV. ASV1 was undetectable in the 3748 sequences of 16S rRNA genes obtained at the beginning of the experiment. At the end, 303 sequences out of 3578 (i.e., 8%) belonged ASV1, while 3578 sequences correspond to 2.47 × 10^8^ copies of 16S rDNA, so we estimated that we had 2.47 × 10^8^ × 303/3578 = 2.09 × 10^7^ copies of *Aeromonas* 16S rDNA at the end of the experiment. For the two instances where the final 16SrDNA abundances were lower than the initial 16S rDNA abundances (possibly due to part of the population dying combined with another part showing small growth), we neglected the contribution of these ASVs to the ^13^C mass balance.

STEP3: We converted the increase in the 16S rDNA from STEP2 into the number of cells using a database that associates a 16S rDNA copy number with each bacterial genus [12]. This database associated ASV1 with 10 copies of 16S rDNA, so the 2.09 × 10^7^ copies of 16S rDNA that were estimated in STEP2 actually correspond to 2.09 × 10^6^ *Aeromonas* cells. It should be noted that the estimated generation time is within a realistic range: ASV 1 was undetectable at t_0_, so if only 1 cell of ASV 1 (*Aeromonas* sp.) was present at the beginning of the experiment, 22 generations would have been needed in the course of the experiment to produce 2 × 10^6^ cells, i.e., 62 min per generation. If ASV 1 was just below the detection limit ((1/3748 × 9.78 × 10^7^)/10 = 2600), the same rationale estimates an average growth rate of 125 min per generation.

STEP4: We converted the number of cells to their carbon content. It should be noted that 30 fg of carbon was measured for the dried *Aeromonas* cells using a Leco CHN analyzer [12], but we assumed the same value for every ASV because the cellular carbon content varied between 20 and 40 fg, depending on the bacterial species. Assuming that each cell contained 30 fg of carbon, we converted the number of cells produced during the course of the experiment to a total carbon reservoir at the end of the experiment (^13^C + ^12^C). *Aeromonas* had 2.09 × 10^7^ cells at the end and a negligible amount at the beginning (undetectable). We then estimated the amount of total carbon that was captured by the growth of ASV1. For example, 2.09 × 10^7^ *Aeromonas* cells translate into 6.28 × 10^−8^ g of total carbon content. 

STEP5: Using the shifts in buoyant density between the ^12^C- and the ^13^C-bottles, we estimated the labeling level of each ASV so that we could estimate their contribution to the ^13^C mass balance. We compared the buoyant density of each ASV in the ^13^C bottle to the density of in the ^12^C bottle by fitting a normal curve to the absolute numbers of the 16S rRNA genes that were detected in each fraction. Since we measured the density of each fraction by refractometry, the mean of the normal curve is the best estimate of the actual buoyant density of the ASV. For example, the 16S DNA of ASV1 have a mean density of 1.72 in the ^12^C bottle and 1.75 in the ^13^C bottle with good fits (R^2^ = 0.98 and 0.89). As mentioned in the text, ASV2 (identified as *Tolumonas* sp.) was not abundant enough in the ^12^C bottle to fit a normal curve on its distribution across the gradient, so we used its theoretical density based on its GC content (which shows a 15% error). The shift between the ^12^C- and ^13^C-densities was converted into a percentage of ^13^C by dividing by 0.036 [13]. The labeling level of ASV1 was (1.75 − 1.72)/0.036 = 85%.

STEP6: To complete the mass balance of the ^13^C-atoms of the labeled T4 phages, we multiplied the total carbon content of each species by its labeling level. For example, ASV1 (*Aeromonas* sp.) represents 6.28 × 10^−8^ × 0.85 = 5.33 × 10^−8^ g of carbon.

STEP7: To estimate the mass of the carbon needed to account for the carbon content observed for each species, we assumed that the carbon use efficiency for each ASV was 33% (see pure culture experiment). It should be noted that we measured the yield for ASV1, and we then we assumed the same yield for every ASV. Once corrected by the carbon use efficiency (i.e., the bacterial yield), the estimation of the ^13^C needed by each species was compared to the 3.2 × 10^−6^ g of ^13^C incorporated in the 2.28 × 10^10 13^C-labeled T4 phages (since each T4 viral particle contained 1.49 × 10^−16^ g C, and we assumed that they were 100% labeled with ^13^C because of their production method). For example, we estimated 2.09 × 10^6^ newly synthesized ASV1 cells, which accounted for 6.28 × 10^−8^ g ^13^C and therefore corresponded to 5% of the ^13^C atoms that were initially present. Adding the contributions of the nine most-labeled ASVs accounted for 41% of the initial mass of ^13^C.

### 2.5. Isolation of Aeromonas_Isolate_007 and Subsequent Experiments

Following the stable isotope probing experiment, we could isolate a strain of *Aeromonas* sp. (corresponding to the amplicon sequence variant ASV1) from the initial sample using the Aeromonas Isolation Agar medium (Sigma 17118) with ampicillin since Aeromonads are resistant to ampicillin. Therefore, we could confirm that *Aeromonas* sp. was indeed able to assimilate the carbon of the T4 phages. Furthermore, we could show that *Aeromonas* could use T4 phages as a carbon and nitrogen source, with a 33% yield, and we also completed a scan the genome of Aeromonas_isolate_007 for the putative mechanisms by which *Aeromonas* could capture and digest the T4 phage proteins and transfer the generated peptides into the intracellular space.

We incubated 50 *Aeromonas* cells with 10^11^ T4 phages in 1mL of SM buffer without gelatin (100 mM NaCl, 8 mM MgSO_4_, 50 mM Tris HCl) at 20 °C to confirm the consumption of T4 phages by *Aeromonas* sp.

### 2.6. Sequencing of Aeromonas_Isolate_007

*Aeromonas* DNA was fragmented by sonication and sequencing adaptors were ligated. A total of eight cycles of PCR were applied to amplify the libraries. Library quality was assessed using an Advanced Analytical Fragment Analyzer, and the libraries were quantified by QPCR using the Kapa Library Quantification Kit. DNA-seq experiments were performed on an Illumina Miseq using a paired-end read length of 2 × 300 pb with the Illumina MiSeq Reagent Kits v3. The sequences were quality trimmed with fastp v0.20.05, assembled by Spades v3.14.16 after removing the residual phiX by using bowtie2 v2.3.5.17 and filtering scaffolds smaller than the right insert size quantile 525 nt and coverage smaller than 50×.

## 3. Results

### 3.1. Stable Isotope Probing Experiment with T4 Bacteriophage

#### 3.1.1. T4 Phages Support Bacterial Growth

To search for bacteriophage consumption activity, we chose wastewater because it contains a high bacterial diversity, high nutrient degradation/turnover rate, and a high microbial metabolic rate. In this work, the stable isotope probing method was applied by adding 2.2 × 10^10 13^C-labelled T4 phages to 200 µL of sludge corresponding to 10^8^ bacteria cells. The enumerated T4 phages decreased by 99% in 24 h (Table 1), at which point the bacterial 16S rDNA genes were analyzed.

The decrease of T4 phages is concomitant with a 2.35-fold increase of the 16S rDNA genes (Table 2). Indeed, the comparison of the 16S and internal standard qPCR curves indicates that the initial sample contained 1.798^23.462^/1.828^13.215^ = 326-fold more bacterial 16S rDNA copies than the internal standard, so the tube contained 3 × 10^5^ × 326 = 9.78 × 10^7^ copies of bacterial 16S at the beginning of the experiment. At the end of the experiment, a comparison of the 16S and internal standard qPCR curves indicates that bacterial 16S rDNA were 783fold more abundant than the spiked synthetic standard, leading to an estimated 2.35 × 10^8^ copies of bacterial 16S rDNA genes in the ^13^C bottle. For an accurate mass balance, we also considered the fact that 240 µL were taken out for PFU measurements during the experiment, so we estimated that 2.47 × 10^8^ copies of 16S rDNA would have been present at the end of the experiment in the ^13^C bottle if no sampling had been performed. Since we used the LinReg software, we also accounted for the slight individual variations in qPCR efficiency, which were between 80 and 84% for the internal standard and between 80 and 82% for the bacterial 16S rRNA genes, respectively. In total, we estimated a global biomass increase of 2.35-fold. This global increase regroups the ASVs that were initially abundant and that have a tendency towards slight growth with the ASVs that are initially rare and grow massively. 

**Figure 1:**
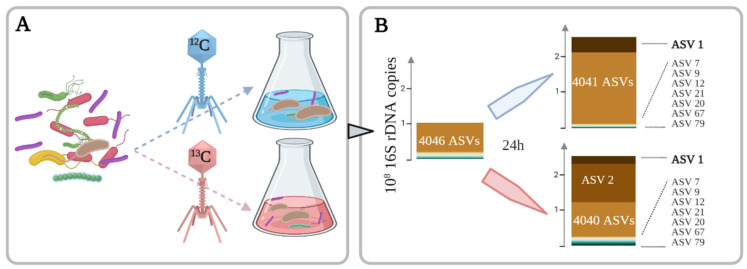
Bacterial growth on T4 phages. (**A**); Identification of ^13^C-labeled bacteria: the ^13^C-labeled T4 (red) were incubated with a microbial community of a wastewater treatment plant in the same conditions as the ^12^C control (blue). (**B**); Bacteria present in each sample: the bar plots show the growth of each ASVs based on the 16S rDNA copies, detailing the nine bacteria assimilating T4 phages.

The bacterial growth concomitant to the decrease of T4 phages changes the composition of the microbial community: In particular ASV1 and ASV2 strongly increased (Figure 1 and Table 3), suggesting that these bacterial species are more adapted to the consumption of bacteriophages, assuming that any other substrates were consumed during the stabilization period before the experiment.

#### 3.1.2. Increase of DNA Density of 9 Microbial Species after 24 h

**Figure 2:**
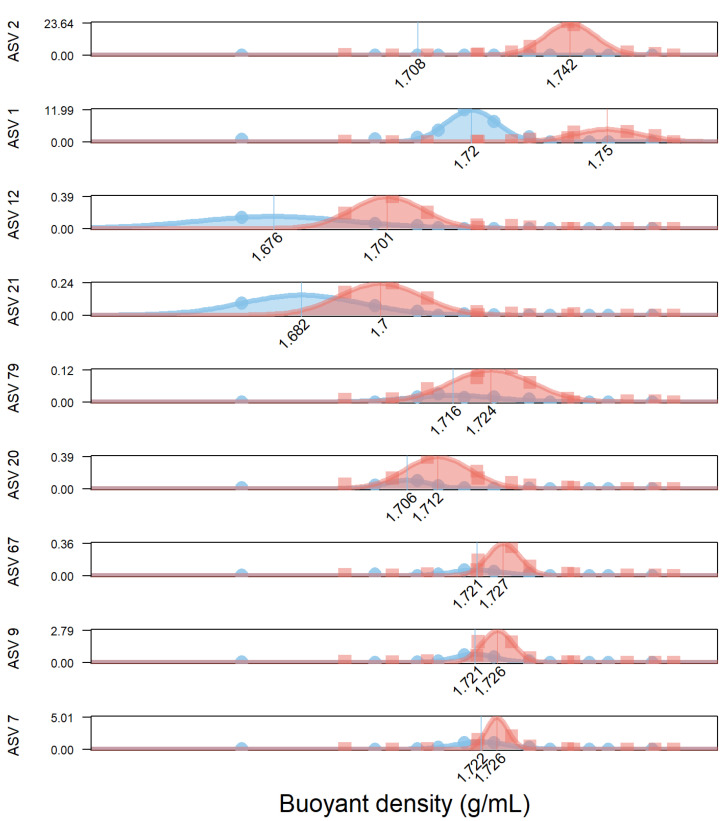
The amount of DNA for the top nine ASVs (in ng) is plotted against the density of the Cesium gradient (in g/mL). The blue color is in the control bottle. The red color indicates the amount of DNA in the bottle supplemented with 3.2 µg of ^13^C in the ^13^C-labeled T4 bacteriophages. The dots indicate the actual measures performed after 24 h, i.e., the amount of DNA of each ASV (Qbit was combined with the 16S rDNA sequencing) in each fraction, the density of which was measured by means of refractometry. The lines indicate the Gaussian distributions to accurately estimate the mean buoyant density. The mean buoyant density of ASV 2 (*Tolumonas* sp.) was estimated with the theoretical value in the ^12^C bottle, as ASV2 did not grow sufficiently in that bottle to fit a reliable Gaussian fit. The pie charts indicate the ratios of the nine ^13^C-labeled ASV in the ^13^C and ^12^C bottles after 24 h, and their proportion in the initial sample is reported in the *X* axis. For example, the pie of Aeromonas is roughly balanced because ASV 1 (*Aeromonas* sp.) represents 19% and 8% in the 12C and 13C bottles, respectively. In contrast, ASV 2 (*Tolumonas* sp.) only grew substantially in the ^13^C bottle (red).

The assimilation of phages by specific members of the bacterial community is confirmed by the increase in the DNA density of nine bacterial species as they assimilate the ^13^C labeled bacteriophages (Figure 2). About 41% of the ^13^C atoms initially present in the T4 phages were accounted for in the bacterial biomass. However, only 9 out of the 4046 microbial species—or more accurately, the Amplicon Sequence Variant (ASVs)—were labelled by the ^13^C initially contained in the T4 phages, thus suggesting that the incorporation of T4 phage is not a widespread ability. This incorporation is in agreement with the 2.35-fold growth and the disappearance of the T4 phages.

The two main degraders of the T4 phages were ASV1 (identified as *Aeromonas* sp.) and ASV2 (identified as *Tolumonas* sp.), which accounted for 5% and 29% of the ^13^C atoms found in the bacterial biomass, respectively. Both belong to the *Aeromonadaceae* family and exhibit strong growth rates. Indeed, both rose from undetectable levels to 51% of the biomass, while the density of their DNA increased because they incorporated ^13^C atoms from the isotopically labelled T4 phages. For example, the 2 × 10^6^ *Aeromonas* cells present after 24 h contained 85% of the ^13^C atoms in their DNA, the density of which shifted from 1.72 g/mL to 1.75 g/mL in the bottle with the ^13^C-labeled T4 phages. The 16S rRNA sequences assigned to *Aeromonas* represented 19% and 8% of the total reads in the ^12^C and the ^13^C bottles, respectively, thus revealing a consistent growth from initially undetectable levels (Figure 1) in addition to the increase of their DNA density (Figure 2).

In addition to the *Aeromonadaceae* family, two species (ASV12 and 21) belonging to the Ignavibacteriales PHOS-HE36 family, although labelled with medium strength (49 and 71%) and negligible growth (0 and 5.87 × 10^6^ synthetized cells, respectively), still gathered 5% of the ^13^C atoms (Table 4). The last five species with significant DNA density shifts (ASV7, 9, 20, 67, and 79) accounted for the remaining 1% of the ^13^C atoms, but their labelling level being below 25% may have resulted from indirect labelling.

### 3.2. Validation in a Pure Culture Experiment

#### 3.2.1. Pure Culture with High Phages Concentrations

**Figure 3:**
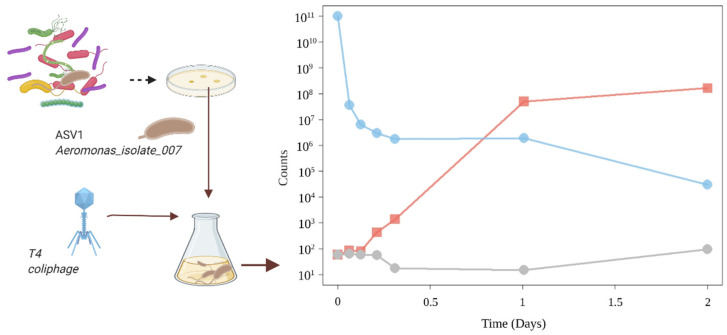
*Aeromonas* sp. growth on T4 phages: Aeromonas_isolate_007 isolated on the Aeromonas Isolation Agar medium grew on T4 phages as the sole carbon and nitrogen source. When a few *Aeromonas* cells were incubated with 10^11^ T4 phages, the colony forming units (red) increased while the plaque-forming units (blue) decreased. The *Aeromonas* cell control in SM buffer without phages (grey) confirms that *Aeromonas* cannot use the Tris from the SM buffer as a carbon source.

To confirm the quality of *Aeromonas* sp. as a predator of T4 phages, an *Aeromonas*-selective medium was used for retrieving an *Aeromonas* colony from the initial sludge. It is called Aeromonas_isolate_007 in the text below. The analysis of the whole genome confirmed that this isolate belongs to an intermediate clade between the *Aeromonas media* and *Aeromonas rivipollensis* species and that it has 10 copies of 16S rDNA based on the coverage ratio. Aeromonas_isolate_007 was incubated with T4 phages only as a substrate. Starting with 50 resting bacterial cells, the population reached 1.63 × 10^8^ cells after 24 h at 20 °C while consuming 10^11^ T4 phages (Figure 3). No growth was observed when the T4 phages were absent from the SM buffer, and no degradation was observed when the bacterial cells were absent.

#### 3.2.2. Pure Culture with Low Phages Concentrations

*Aeromonas* sp. could also capture T4 phages when their concentrations were comparable with environmental conditions: 7 × 10^4^ T4 phages/mL decreased to 2 × 10^3^ T4 phages/mL when incubated with Aeromonas_isolate_007 cells. No decrease in the T7 phages have been observed in similar experiments where the T4 phages were replaced by T7 phages (data not shown).

#### 3.2.3. Sequencing Aeromonas_Isolate_007

**Figure A1:**
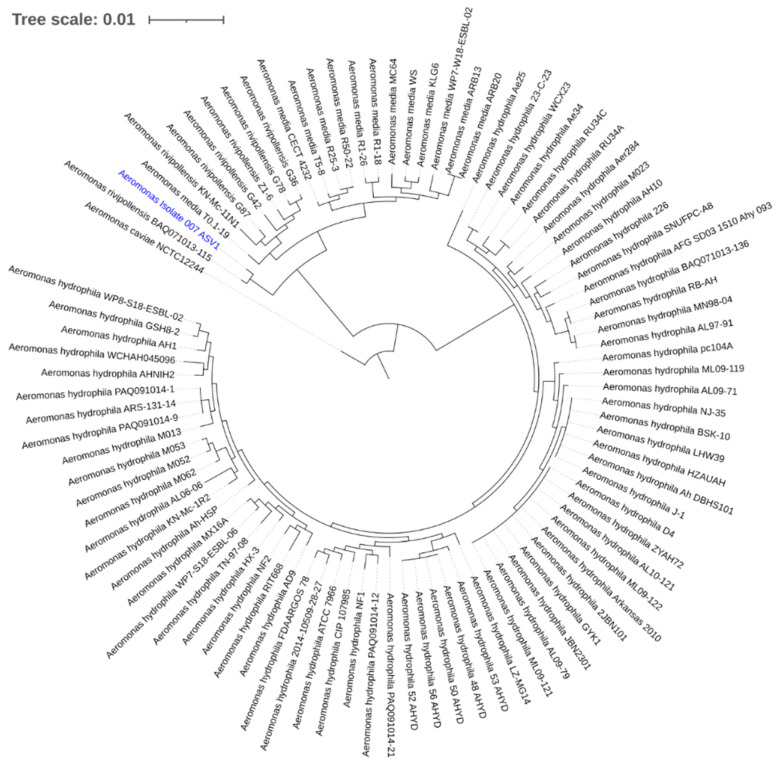
Phylogenetic tree of Aeromonas_Isolate_007_151020.

**Figure A2:**
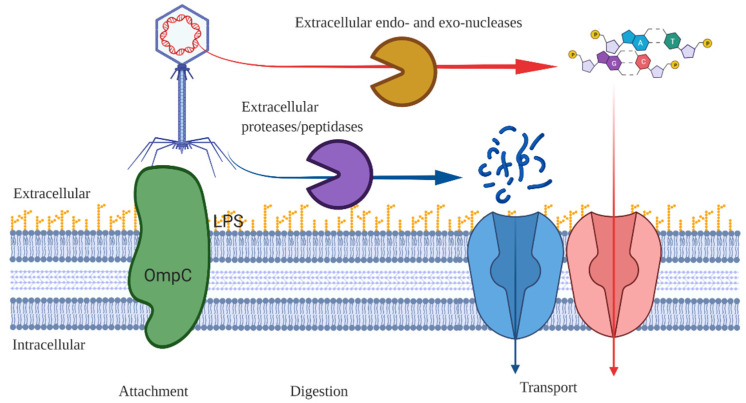
Schematic representation of the capture, digestion, and absorption of the T4 phages by *Aeromonas* sp. Since T4 phages are large particles (not to scale in the figure), we assume that *Aeromonas* captures the T4 phages due to its similarity with the cell wall of *E. coli*. Once captured, the proteins of the capsid may be digested by the extracellular proteases present in *Aeromonas* sp. and the DNA could be degraded as well. The products of these degradations could be ingested by *Aeromonas* via common transporters.

**Figure A3:**
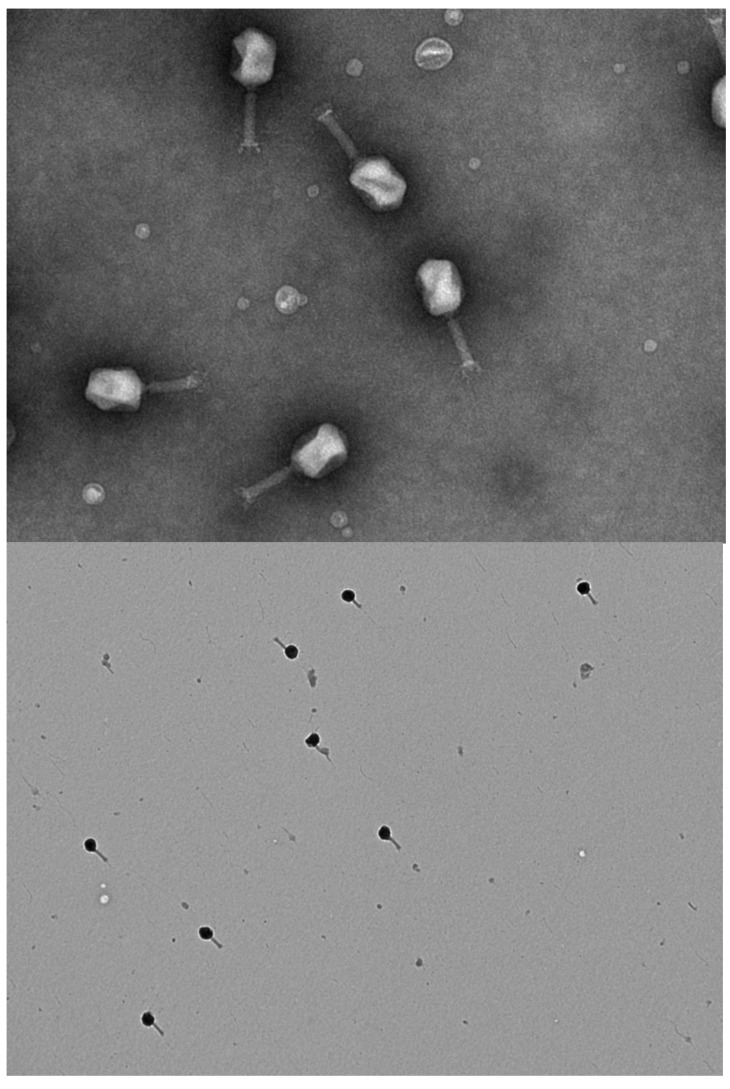
Purity of the bacteriophage preparation. We checked the purity of the bacteriophage preparation by means of electronic microscopy. Materials were directly adsorbed onto a carbon film membrane on a 300-mesh copper grid, stained with 1% uranyl acetate, dissolved in distilled water, and dried at room temperature. Grids were examined with a Hitachi HT7700 electron microscope operated at 80kV (Elexience—France), and the images were acquired with a charge-coupled device camera (AMT).

Aeromonas_isolate_007 had a genome of 4,667,413 nt in 29 scaffolds with a N50 of 947,468 nt and an average size of 160,945 nt. This represents 4172 genes, and the number of 16S rRNA copies was estimated to be 10.89 with the coverage, which is in accordance with the Vetrovsky database. The whole genome sequencing of Aeromonas_isolate_007 by Illumina Miseq narrows down the phylogeny of the strain (Appendix A Figure A1) and offers suggestions with respect to degradative enzymes that may help bacteriophage digestion (Appendix A Figure A2).

## 4. Discussion

*Aeromonas* cells are widely distributed [15], including in wastewater treatment plants, where their abundance is around 0.1% [16].

Interestingly, *Aeromonas* cells have an S-layer [17] that is associated with lipopolysaccharides [18] and an outer membrane protein C (Appendix A Figure A2 and Appendix B Table A1), which are known to bind the T4 phages to the surface of *E.coli* cells [19]. Once captured at the surface, the phage is likely degraded by several extracellular enzymes, including DNase and protease [20]. For example, metallo- and serine-proteases found in *Aeromonas* are involved in the degradation of large molecules such as albumin, earning the nickname of “Jack-of-all-trades” due to this enzymatic versatility [21]. Finally, *Aeromonas* possesses transporters to uptake the resulting amino acids and peptides [21].

Bacterial predation on bacteriophages is rich in consequences because bacteriophages can control the abundance of specific bacterial species. Indeed bacteriophage decay is mainly considered abiotic via adhesion to particulate material, chemical inactivation, or degradation by solar radiation or passive grazing by flagellates [6]. Here, we showed that there might be a non-negligible fraction of phage degradation that could be due to low-abundant bacterial species.

Furthermore, the diversity in bacteriophages could be partly related to the presence of phage-specific bacterial predation. Indeed, the bacterial predators of T4 phages do not appear to consume T7 bacteriophages. Therefore, a rapid increase of a specific phage in the environment could be specifically controlled by a phage-eating bacterium, forming a killing-the-killer loop. This is especially true as the two main degraders in our experiment were initially undetectable. Therefore, the control of the T4 phages at the end of our experiment is likely to be more pronounced than at the beginning of the experiment, thereby potentially changing the dynamics of *E. coli* and T4 if T4 was controlling *E. coli* in our environment.

In conclusion, specific bacteria that are capable of eliminating specific viruses changes our vision of food webs and represent a noteworthy avenue to explore to control harmful bacteriophages that disrupt dairy fermentation.

## Figures and Tables

**Table 1:** Decrease of the free ^13^C and ^12^C bacteriophages by PFU of the supernatant. We indicate the absolute numbers of phages and their percentages compared to t0.

Time (min)	T4 Phages in ^12^C Bottle	T4 Phages in ^13^C Bottle	T4 Phages in ^12^C Bottle	T4 Phages in ^13^C Bottle
0	2.8 × 10^10^	2.2 × 10^10^	100%	100%
24	5.2 × 10^8^	9.4 × 10^8^	1.9%	4.2%
122	2.6 × 10^7^	4.1 × 10^8^	0.9%	1.8%
445	3.2 × 10^7^	3.3 × 10^8^	1.2%	1.5%
1375	5 × 10^2^	5.7 × 10^5^	0%	0%

**Table 2:** Quantification of the bacterial density with the internal standard at the beginning and at the end of the experiment.

Sample Name	Primers	Efficiency	Cycle Threshold	Spiked Internal Standard	16S rDNA
Initial sample	Internal standard	79.8%	23.462	3 × 10^5^ copies	9.78 × 10^7^ copies
	V4V5	82.8%	13.215		
Final ^13^C sample	Internal standard	80.1%	14.133	3 × 10^5^ copies	2.35 × 10^8^ copies
	V4V5	84.5%	24.461		

**Table 3:** Taxonomic affiliation of the ASVs that are significantly labeled with ^13^C. This table indicates the taxonomy of the labeled ASV performed by DADA2 with the Silva138 dataset. The taxonomy was also checked by means of blasting on the NCBI database.

seq_ID.x	Class	Order	Family	Genus
ASV 2	Gammaproteobacteria	Aeromonadales	Aeromonadaceae	*Tolumonas*
ASV 1	Gammaproteobacteria	Aeromonadales	Aeromonadaceae	*Aeromonas*
ASV 12	Ignavibacteria	Ignavibacteriales	PHOS-HE36	NA
ASV 21	Ignavibacteria	Ignavibacteriales	PHOS-HE36	NA
ASV 79	Bacteroidia	Chitinophagales	Saprospiraceae	*Haliscomenobacter*
ASV 20	Bacteroidia	Chitinophagales	Saprospiraceae	NA
ASV 67	Anaerolineae	Ardenticatenales	NA	NA
ASV 7	Gammaproteobacteria	Burkholderiales	Rhodocyclaceae	NA
ASV 9	Gammaproteobacteria	Burkholderiales	Rhodocyclaceae	*Dechloromonas*

**Table 4:** ^13^C mass balance of the isotopically labeled T4 bacteriophages. This table indicates the rationale for the ^13^C mass balance following the steps described above. For example, ASV 1 (*Aeromonas* sp.) represents 8% of the 2.47 × 10^8^ 16S rDNA copies found at the end of the experiment, which represents 5% of the total amount of 13C present in the initial T4 phages because the 30fgC-cells labeled at 85% needed 5.3 × 10^−8^ g of 13C if we consider a 33% yield.

	Description	ASV 1	ASV 2	ASV 7	ASV 9	ASV 12	ASV 20	ASV 21	ASV 67	ASV 79
STEP 1	Absolute number of 16S rDNA copies in the initial sample	9.78 × 10^7^								
	Absolute number of 16S rDNA copies in the final sample	2.47 × 10^8^								
STEP 2	Counts in the initial sample (out of 3748 sequences)	0	0	148	132	197	123	0	46	53
	Counts in the final ^13^C sample (out of 3578 sequences)	303	1545	37	62	52	55	55	41	55
	Relative initial abundance of 16S rDNA of each ASV	0%	0%	4%	4%	5%	3%	0%	1%	1%
	Relative final abundance of 16S rDNA of each ASV	8%	43%	1%	2%	1%	2%	2%	1%	2%
	Absolute number of 16S rDNA copies in the initial sample of each ASV	0	0	3.86 × 10^6^	3.44 × 10^6^	5.14 × 10^6^	3.21 × 10^6^	0	1.20 × 10^6^	1.38 × 10^6^
	Absolute number of 16S rDNA copies in the final sample of each ASV	2.09 × 10^7^	1.07 × 10^8^	2.55 × 10^6^	4.28 × 10^6^	3.59 × 10^6^	3.80 × 10^6^	3.80 × 10^6^	2.83 × 10^6^	3.80 × 10^6^
	Number of newly synthetized 16S copies	2.09 × 10^7^	1.07 × 10^8^	0	8.36 × 10^5^	0	5.87 × 10^5^	3.80 × 10^6^	1.63 × 10^6^	2.41 × 10^6^
STEP 3	Number of 16S rDNA copies per genome of each ASV [14]	10	10	4	4	1	2	1	2	2
	Number of newly synthetized cells of each ASV	2.09 × 10^6^	1.07 × 10^7^	0	2.09 × 10^5^	0	2.94 × 10^5^	3.80 × 10^6^	8.15 × 10^5^	1.21 × 10^6^
STEP 4	Carbon content (g/cell)	3.00 × 10^−14^								
	Total Carbon content in each ASV (g)	6.28 × 10^−8^	3.20 × 10^−7^	0	6.27 × 10^−9^	0	8.81 × 10^−9^	1.14 × 10^−7^	2.45 × 10^−8^	3.62 × 10^−8^
STEP 5	Mean 12C density (g/mL)	1.72	1.7	1.72	1.72	1.68	1.71	1.68	1.72	1.72
	Goodness_fit_in_^12^C (R^2^)	0.98	0.4	0.99	0.97	0.99	0.97	0.98	0.88	0.89
	Mean corrected ^12^C density	1.72	1.71	1.72	1.72	1.68	1.71	1.68	1.72	1.72
	Mean ^13^C density	1.75	1.74	1.73	1.73	1.7	1.71	1.7	1.73	1.72
	Goodness_fit_in_^13^C (R^2^)	0.89	0.99	0.9	0.89	0.98	0.93	0.99	0.94	0.94
	Labeling Level (%)	85%	95%	10%	14%	71%	19%	49%	16%	23%
STEP 6	^13^C carbon content in each ASV (g)	5.3 × 10^−8^	3.0 × 10^−7^	0	8.8 × 10^−10^	0	1.7 × 10^−9^	5.6 × 10^−8^	3.9 × 10^−9^	8.3 × 10^−9^
STEP 7	Carbon use efficiency (i.e., Bacterial yield)	0.33								
	Contribution to the ^13^C mass balance (out of the 3.2 µg of ^13^C in the bacteriophages)	5% ^1^	29% ^1^	0%^1^	0% ^1^	0%	0%	5%	0%	1%
	^13^C mass balance	41%								

^1^, 83% of the predation of T4 phages is due to *Gammaproteobacteria*, which also include *E. coli*, the natural host of T4 phages.

## Data Availability

High-throughput sequencing data have been deposited on NCBI (https://www.ncbi.nlm.nih.gov/bioproject) (accessed on 11 August 2021)under accession number PRJNA650397, and the genome of Aeromonas_isolate_007 is accessible with the BioSample accession number SAMN17689348.

## Appendix A

The analysis of the whole genome confirms that Aeromonas_Isolate_007_151020 belongs to an intermediate clade between the *Aeromonas media* and *Aeromonas rivipollensis* species.

## Appendix B

The functional annotation was performed with RAST8, eggNOG-mapper (v2.0.0)9 and TMHMM10 to predict the extracellular location of the proteins. The results are reported in the table below.

**Table A1:** Annotation of the *Aeromonas* proteins putatively involved in the capture, digestion, and absorption of T4 phages, which are reported in the table below.

Putative Function	RAST Protein Id	Contig Id	RAST_Function	TMHMM Inside	TMHMM Transmembrane	TMHMM Outside
CAPTURE	fig|642.770.peg.2774	NODE_2_length_947468_cov_91.192050	Outer_membrane_porin_OmpC	1	1	1
	fig|642.770.peg.1967	NODE_1_length_1964260_cov_90.024368		0	0	1
	fig|642.770.peg.3181	NODE_3_length_439005_cov_94.027299		0	0	1
Extracellular DIGESTION of large proteins	fig|642.770.peg.202	NODE_1_length_1964260_cov_90.024368	putative_extracellular_serine_protease	0	0	1
	fig|642.770.peg.309	NODE_1_length_1964260_cov_90.024368	Uncharacterized_protease_YhbU	0	0	1
	fig|642.770.peg.653	NODE_1_length_1964260_cov_90.024368	Tail-specific_protease_precursor_(EC_3.4.21.102)	0	0	1
	fig|642.770.peg.664	NODE_1_length_1964260_cov_90.024368	Lon_protease_homolog_YcbZ	0	0	1
	fig|642.770.peg.903	NODE_1_length_1964260_cov_90.024368	ATP-dependent_protease_La_(EC_3.4.21.53)_Type_I	0	0	1
	fig|642.770.peg.904	NODE_1_length_1964260_cov_90.024368	ATP-dependent_Clp_protease_ATP-binding_subunit_ClpX	0	0	1
	fig|642.770.peg.905	NODE_1_length_1964260_cov_90.024368	ATP-dependent_Clp_protease_proteolytic_subunit_ClpP_(EC_3.4.21.92)	0	0	1
	fig|642.770.peg.919	NODE_1_length_1964260_cov_90.024368	Protease_III_precursor_(EC_3.4.24.55)	0	0	1
	fig|642.770.peg.976	NODE_1_length_1964260_cov_90.024368	ATP-dependent_Clp_protease_ATP-binding_subunit_ClpA	0	0	1
	fig|642.770.peg.1312	NODE_1_length_1964260_cov_90.024368	Protease_II_(EC_3.4.21.83)	0	0	1
	fig|642.770.peg.1793	NODE_1_length_1964260_cov_90.024368	Uncharacterized_protease_YegQ	0	0	1
	fig|642.770.peg.1989	NODE_1_length_1964260_cov_90.024368	Vibriolysin__extracellular_zinc_protease_(EC_3.4.24.25)_@_Pseudolysin__extracellular_zinc_protease_(EC_3.4.24.26)	0	0	1
	fig|642.770.peg.2147	NODE_2_length_947468_cov_91.192050	Uncharacterized_protease_YdcP	0	0	1
	fig|642.770.peg.3280	NODE_3_length_439005_cov_94.027299	Protease_II_(EC_3.4.21.83)	0	0	1
	fig|642.770.peg.3795	NODE_5_length_206066_cov_93.597574	ATP-dependent_hsl_protease_ATP-binding_subunit_HslU	0	0	1
	fig|642.770.peg.3796	NODE_5_length_206066_cov_93.597574	ATP-dependent_protease_subunit_HslV_(EC_3.4.25.2)	0	0	1
	fig|642.770.peg.12	NODE_10_length_72653_cov_95.226534	Oligopeptidase_A_(EC_3.4.24.70)	0	0	1
	fig|642.770.peg.67	NODE_10_length_72653_cov_95.226534	Xaa-Pro_dipeptidase_PepQ_(EC_3.4.13.9)	0	0	1
	fig|642.770.peg.308	NODE_1_length_1964260_cov_90.024368	Uncharacterized_peptidase_U32_family_member_YhbV	0	0	1
	fig|642.770.peg.319	NODE_1_length_1964260_cov_90.024368	Peptidase_B_(EC_3.4.11.23)	0	0	1
	fig|642.770.peg.324	NODE_1_length_1964260_cov_90.024368	Peptidase_B_(EC_3.4.11.23)	0	0	1
	fig|642.770.peg.496	NODE_1_length_1964260_cov_90.024368	Aminopeptidase_PepA-related_protein	0	0	1
	fig|642.770.peg.654	NODE_1_length_1964260_cov_90.024368	Membrane_alanine_aminopeptidase_N_(EC_3.4.11.2)	0	0	1
	fig|642.770.peg.1189	NODE_1_length_1964260_cov_90.024368	Oligoendopeptidase_F-like_protein	0	0	1
	fig|642.770.peg.1360	NODE_1_length_1964260_cov_90.024368	Tripeptide_aminopeptidase_(EC_3.4.11.4)	0	0	1
	fig|642.770.peg.1462	NODE_1_length_1964260_cov_90.024368	Probable_endopeptidase_NlpC	0	0	1
Extracellular DIGESTION of large proteins	fig|642.770.peg.1502	NODE_1_length_1964260_cov_90.024368	FIG009095:_D_D-carboxypeptidase_family_protein	0	0	1
	fig|642.770.peg.1638	NODE_1_length_1964260_cov_90.024368	Peptidase__M23/M37_family	0	0	1
	fig|642.770.peg.1722	NODE_1_length_1964260_cov_90.024368	Membrane_proteins_related_to_metalloendopeptidases	0	0	1
	fig|642.770.peg.1792	NODE_1_length_1964260_cov_90.024368	L_D-transpeptidase_>_YbiS	0	0	1
	fig|642.770.peg.1981	NODE_1_length_1964260_cov_90.024368	L_D-transpeptidase_>_YbiS	0	0	1
	fig|642.770.peg.2129	NODE_2_length_947468_cov_91.192050	Alpha-aspartyl_dipeptidase_Peptidase_E_(EC_3.4.13.21)	0	0	1
	fig|642.770.peg.2153	NODE_2_length_947468_cov_91.192050	Thermostable_carboxypeptidase_1_(EC_3.4.17.19)	0	0	1
	fig|642.770.peg.2266	NODE_2_length_947468_cov_91.192050	Methionine_aminopeptidase_(EC_3.4.11.18)	0	0	1
	fig|642.770.peg.2291	NODE_2_length_947468_cov_91.192050	Peptidase__M13_family	0	0	1
	fig|642.770.peg.2456	NODE_2_length_947468_cov_91.192050	γ-glutamyltranspeptidase_(EC_2.3.2.2)_ @_Glutathione_hydrolase_(EC_3.4.19.13)	0	0	1
	fig|642.770.peg.3506	NODE_4_length_411610_cov_92.800192	Xaa-Pro_aminopeptidase_(EC_3.4.11.9)	0	0	1
	fig|642.770.peg.3576	NODE_4_length_411610_cov_92.800192	Prolyl_endopeptidase_(EC_3.4.21.26)	0	0	1
	fig|642.770.peg.4017	NODE_6_length_154685_cov_89.330397	Oligoendopeptidase_F-like_protein	0	0	1
	fig|642.770.peg.4147	NODE_7_length_126480_cov_93.962673	Bacterial_leucyl_aminopeptidase_(EC_3.4.11.10)	0	0	1
DNA DIGESTION	fig|642.770.peg.760	NODE_1_length_1964260_cov_90.024368	Extracellular_and/or_outer_membrane_deoxyribonuclease_NucH/SO1066	0	0	1
	fig|642.770.peg.1409	NODE_1_length_1964260_cov_90.024368	UPF0294_protein_YafD (exo- and endo- nuclease family)	0	0	1
	fig|642.770.peg.1884	NODE_1_length_1964260_cov_90.024368	DNA/RNA_endonuclease_G	1	1	1
	fig|642.770.peg.1925	NODE_1_length_1964260_cov_90.024368	Extracellular_and/or_outer_membrane_deoxyribonuclease_NucH/SO1066	0	0	1
Peptide TRANSPORT into the cell	fig|642.770.peg.2135	NODE_2_length_947468_cov_91.192050	Succinyl-CoA_synthetase__alpha_subunit	0	0	1
	fig|642.770.peg.274	NODE_1_length_1964260_cov_90.024368	ABC_transporter__permease_protein_1_(cluster_5__nickel/peptides/opines)	4	6	3
	fig|642.770.peg.275	NODE_1_length_1964260_cov_90.024368	ABC_transporter__permease_protein_2_(cluster_5__nickel/peptides/opines)	3	5	3
	fig|642.770.peg.1213	NODE_1_length_1964260_cov_90.024368	Oligopeptide_ABC_transporter__permease_protein_OppC_(TC_3.A.1.5.1)	4	6	3
	fig|642.770.peg.1214	NODE_1_length_1964260_cov_90.024368	Oligopeptide_ABC_transporter__permease_protein_OppB_(TC_3.A.1.5.1)	4	6	3
	fig|642.770.peg.1215	NODE_1_length_1964260_cov_90.024368	Oligopeptide_ABC_transporter__substrate-binding_protein_OppA_(TC_3.A.1.5.1)	1	1	1
	fig|642.770.peg.1819	NODE_1_length_1964260_cov_90.024368	Dipeptide_ABC_transporter__permease_protein_DppC_(TC_3.A.1.5.2)	4	6	3
	fig|642.770.peg.1820	NODE_1_length_1964260_cov_90.024368	ABC_transporter__permease_protein_1_(cluster_5__nickel/peptides/opines)	4	6	3
	fig|642.770.peg.1913	NODE_1_length_1964260_cov_90.024368	ABC_transporter__permease_protein_2_(cluster_5__nickel/peptides/opines)	4	6	3
	fig|642.770.peg.1914	NODE_1_length_1964260_cov_90.024368	ABC_transporter__permease_protein_1_(cluster_5__nickel/peptides/opines)	4	6	3
DNA TRANSPORT into the cell	fig|642.770.peg.2922	NODE_3_length_439005_cov_94.027299	Na+_dependent_nucleoside_transporter_NupC	5	9	4
	fig|642.770.peg.2967	NODE_3_length_439005_cov_94.027299	Na+_dependent_nucleoside_transporter_NupC	4	8	4
	fig|642.770.peg.3112	NODE_3_length_439005_cov_94.027299	Na+_dependent_nucleoside_transporter_NupC	4	8	4
	fig|642.770.peg.4145	NODE_7_length_126480_cov_93.962673	Predicted_nucleoside_ABC_transporter__permease_1_component	6	10	5
	fig|642.770.peg.4146	NODE_7_length_126480_cov_93.962673	Predicted_nucleoside_ABC_transporter__permease_2_component	4	7	4
	fig|642.770.peg.177	NODE_13_length_22868_cov_91.969299	DNA_uptake_protein	0	0	1

## Appendix C

Appendix C describes the purity of the initial solution of T4 phages.

T4-phage particles labeled with ^13^C were produced on *Escherichia coli* B cells (DSM 613) grown in M9 minimal medium with ^13^C-glucose as the sole carbon source. The M9 medium was prepared using M9, Minimal Salts, 5× (Sigma-Aldrich) by adding MgSO_4_ (Sigma-Aldrich) and CaCl_2_ (Sigma-Aldrich) at final concentrations of 1 mM and D-Glucose at a final concentration of 10 g/L. Moreover, additional salts were added to favor phage adsorption (solution of CaCl_2_ 0.5 M and MgCl_2_ 1M, diluted 1 000 times in the culture medium). More precisely, starting from an *E. coli* stock of cells frozen in LB and glycerol, two successive overnight pre-cultures were grown in LB medium (LB broth, Fisher, Camas, WA, USA). Subsequently, 5 × 20 mL of M9 minimal medium containing D-Glucose-^13^C6 as the sole carbon source (10 g/L) were each inoculated with 20 µL of the second *E. coli* pre-culture; approximately 1500 T4-phage particles (DSM 4505, in PFU) were added. Finally, 20 µL of a solution containing 0.5 M CaCl_2_ and 1 M MgCl_2_ were also added in each case to favor phage adsorption.

After 30 h of incubation at 37 °C under 50 rpm agitation to allow the phages to replicate, the T4 phage particles were collected: the cultures were centrifuged for 15 min at 5000× *g* at 10 °C to eliminate the large cellular debris. The supernatants were collected and filtered through 0.22 µm pore-sized PES filters (Milipore, Burlington, MA, USA) to eliminate fine cellular debris. They were subsequently incubated overnight in 8% *w/v* PEG 6000 and 0.5 M NaCl solution at 4 °C to aggregate the viral particles with minimal losses. The phage aggregates were collected by centrifugation at 20,000× *g* for 30 min at 4 °C. The pellets were resuspended in SM buffer (100 mM NaCl, 8 mM MgSO_4_, 50 mM Tris pH 7.5), and were centrifuged once more at 20,000× *g* for 4h at 4 °C. The viral particles were finally suspended in 1.4 mL of SM buffer and were stored at 4 °C before use.

To obtain the unlabeled T4 phage particles, the same procedure was used, except unlabeled glucose was employed in the M9 minimal medium.
